# Folate-conjugated organic CO prodrugs: Synthesis and CO release kinetic studies

**DOI:** 10.21203/rs.3.rs-4213303/v1

**Published:** 2024-04-12

**Authors:** Shameer M. Kondengadan, Shubham Bansal, Xiaoxiao Yang, Binghe Wang

**Affiliations:** Georgia State University; Georgia State University; Georgia State University; Georgia State University

**Keywords:** Enrichment triggered delivery, Carbon Monoxide, Folate conjugate, Reaction kinetics, Diels-Alder reaction

## Abstract

Carbon monoxide (CO) is an endogenous produced molecule and has shown efficacy in animal models of inflammation, organ injury, colitis and cancer metastasis. Because of its gaseous nature, there is a need for developing efficient CO delivery approaches, especially those capable of targeted delivery. In this study, we aim to take advantage of a previously reported approach of enrichment-triggered prodrug activation to achieve targeted delivery by targeting the folate receptor. The general idea is to exploit folate receptor-mediated enrichment as a way to accelerate a biomolecular Diels-Alder reaction for prodrug activation. In doing so, we first need to find ways to tune the reaction kinetics in order to ensure minimal rection without enrichment and optimal activation upon enrichment. In this feasibility study, we synthesized two diene-dienophile pairs and studied their reaction kinetics and ability to target the folate receptor. We found that folate conjugation significantly affects the reaction kinetics of the original diene-dienophile pairs. Such information will be very useful in future designs of similar targeted approaches of CO delivery.

## 1 Introduction

Success of conventional therapeutic approach is often limited by the off-target drug delivery and associated side-effects.[1, 2] To a great extent, such kind of off-target effects can be minimized by targeted delivery of drugs to the intended site, sparing the healthy cells.[3, 4, 2] In general, pathophysiology of most disease is often associated with over expression of certain biomarkers,[5, 6] providing an opportunity for targeted delivery of therapeutics. Folic acid, also known as vitamin-B_9_, is essential for cell growth and proliferation by helping DNA and RNA synthesis.[7, 8] Because mammals do not synthesize folate, it has to come from nutrients. Further, cellular uptake is essential for all types of cells for their routine metabolic process, and the demand is generally high in rapidly metabolizing tumor cells. Generally, folate uptake in mammalian cells happens through three different mechanisms, a proton coupled folate transporter, reduced folate receptor carrier (RFC), and folate receptors (FRs).[9] FRs are mainly enriched in tumor cells, macrophages, and proximal tubule cells, whereas the former two pathways are ubiquitous in nature.[10] FR is a glycosylphosphatidyl inositol modified cell-surface receptor and uptakes the folate via receptor mediated endocytosis.[11] Literature reports indicate high affinity binding by FRs (K_d_ ~ 10^− 9^ M);[12] thus, conjugation with folic acid provides an efficient approach for the targeted delivery of payloads to FR expressing to proximal tubule cells and tumor cells as well as inflamed tissues such as in colitis.[13] Folate receptors (FRs) are of four different isoforms: FRα, FRβ, FRγ and FRδ. Among these, FRα, FRβ, and FRδ are anchored by cell-surface GPI proteins, whereas FRδ lacks the GPI proteins and consequently functions as a secretory protein.[9] We are interested in ways for intracellular delivery of carbon monoxide (CO) via folate receptor-mediated uptake for treating organ injury such as acute kidney injury[14] and inflammatory conditions such as colitis.[15]

**Figure 1:**
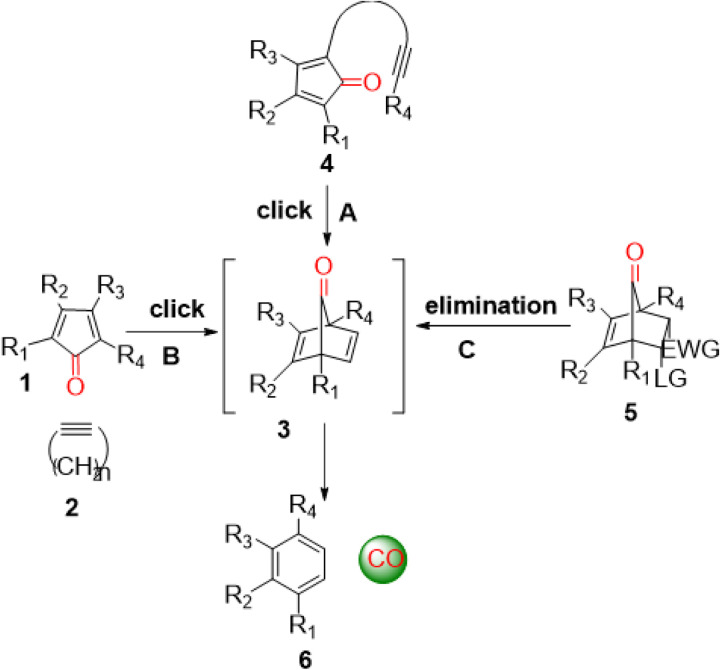
Design of organic CO prodrugs.[53]

Carbon monoxide (CO) is endogenously produced as part of red blood cell turn over with significance on par with nitric oxide and hydrogen sulfide.[16] CO has shown pharmacological effects in animal models of systemic inflammation,[17, 18] lung injury,[19] liver injury,[20, 21] kidney injury,[22, 23] colitis,[15] and cancer metastasis.[24] Aimed at searching for easy, safe, and controllable delivery of CO as alternatives to inhaled CO gas, many labs have worked on CO donors of various types including metal-carbonyl complexes as CO-releasing molecules (CORMs)[25–28] and CORMs for triggered CO release.[29–33] Beyond metal-carbonyl complexes, there are organic CO donors that are photo-sensitive, ROS-sensitive, mechanical force-sensitive, or chemoexcitation-sensitive.[34–40] There are also innovative ways of trapped CO,[41] CO in micelles,[29, 42] and CO solution.[43, 41] In 2014, we reported the first organic CO prodrugs by taking advantage of a cheletropic reaction for CO release from a norbornadienone scaffold (**3**, Fig. 1), which can be generated by using an intra-molecular Diels-Alder reaction (Fig. 1A),[15, 44] an inter-molecular Diels-Alder reaction (Fig. 1B),[45, 46] or an elimination reaction (Fig. 1C)[47–49] to form the norbornadiene-7-ones intermediate for subsequent CO release (Fig. 1). Independently, the Larsen lab also developed a similar approach of using the norbornadienone scaffold, generated through an elimination reaction.[50–52]

**Figure 2:**
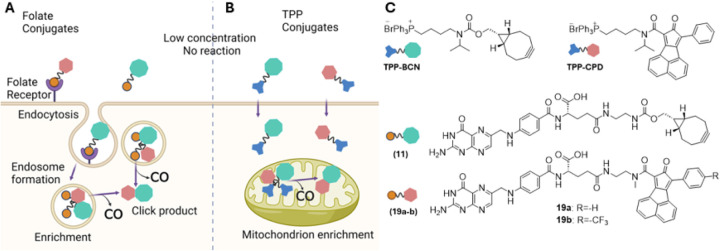
Enrichment triggered delivery of CO by folate (A), and TPP (B) conjugates. C) Structures of folate and TPP conjugates used in Fig. A-B (“Created with BioRender.com”)

Even with all the advances in developing new delivery forms of CO, there is a need for targeted delivery to minimize side effects and improve the safety margin. Along this line, we have worked on CO prodrugs capable of triggered release using stimuli such as esterase,[54, 18] ROS,[48] and pH.[47] Especially worth noting is our efforts on enrichment-triggered release, which allows for targeted delivery of CO with improved potency.[45] The basic design principle relies on the use of a bimolecular approach (Fig. 1B) to generate the norbornadienone scaffold and the concentration-dependent nature of such a biomolecular reaction rate for substantially improved CO release rate upon enrichment. However, past enrichment efforts relied on triphenylphosphonium (TPP)-based mitochondria targeting (Fig. 2B). Though the enrichment part worked well, the use of a bulky hydrophobic TPP group brings in problems of drug-like properties. In this study, we examine the feasibility of using folate-mediated endocytosis as a way to achieve enrichment of the prodrug components and then selective targeting of CO (Fig. 2B). A critical requirement for success in such an enrichment-triggered release approach is the ability to tune the reaction rate in such a way that the bimolecular reaction is slow at dosing concentration and is fast after enrichment. Therefore, it is important to understand how conjugation with folate would affect the reaction rate of the intended bimolecular Diels-Alder reaction (Fig. 1B). In this study, we describe the synthesis of two diene-dienophile pairs that allowed us to achieve a basic understanding of how such conjugation affects reaction rate. Such information will be important for future optimization work.

## 2 Results and Discussion

### Design.

Again, the basic design for the compounds in this study is based on a bimolecular Diels-Alder reaction, leading to the formation of an unstable norbornadiene-7-one intermediate with subsequent spontaneous CO release through a cheletropic extrusion reaction (Fig. 1B).

**Figure 3:**
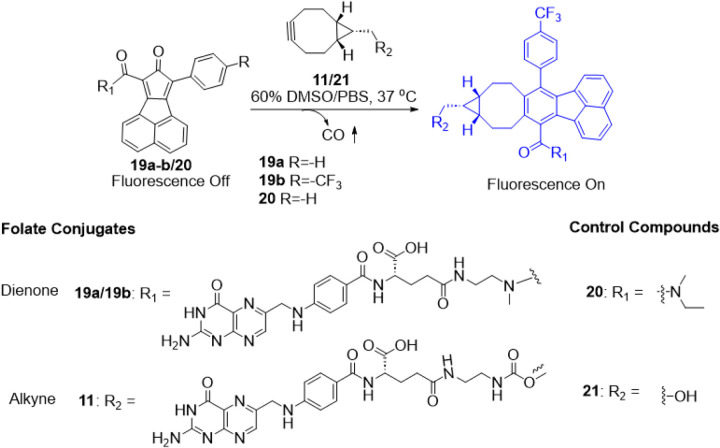
CO release kinetic studies of folate-conjugated prodrug partners (**19a/19b, 11**), and control compounds (**20, 21**) by using turn-on fluorescence of the product.

Specifically, norbornadiene-7-ones (Fig. 1, 3) are highly unstable under physiological conditions and undergo a spontaneous bond-breaking cheletropic reaction to release CO. By using an inter-molecular Diels-Alder reaction, our lab has demonstrated enrichment-triggered delivery of CO inside the mitochondria.[45] In this bimolecular reaction, both reaction partners, cyclopentadienone (CPD) and strained cycloalkyne (BCN), are conjugated with TPP for enrichment in mitochondria (Fig. 2B). A key aspect of the research design was to tune the reaction rate in such a way that at the concentration of systemic dosing, the reaction has a long half-life. However, reaction half-life would shorten proportionally upon enrichment in mitochondria, leading to quick CO release. Because TPP-mediated enrichment is known to reach several hundred-fold, the reaction rate constant can be tuned accordingly. Specifically, the reaction rate constant between TPP-BCN and TPP-CPD (Fig. 2C) was tuned to about 0.20 M^− 1^s^− 1^, which gives a calculated t_1/2_ of 139 h at 10 μM. Because mitochondria-enrichment is known to reach hundreds of folds, after enrichment, the reaction rate can also accelerate by hundreds of folds to allow CO release with a t_1/2_ of a few hours or less. We were indeed able to demonstrate proof of principle using cell culture and animal models.[45] Then, we are interested in moving away from using the hydrophobic TPP moiety for enrichment. Thus, we turn to folate-receptor-mediated enrichment for triggered release and targeted release in cells that over-express such receptor.

First, we are interested in searching for treatment options for AKI and colitis. Folate receptor is known to be enriched in proximal tubule cells, offering a site for targeted delivery.[10] Second, macrophages are known to over express the folate receptor,[10] allowing for targeted delivery of a payload to inflamed tissues including colitis. Therefore, we sought to synthesize conjugates of folate with the two respective reaction components (Fig. 2C) and assess their reactivity (reaction kinetics).

### 2.1 Synthesis of folate conjugated cyclopentadienone and cyclooctyne

In the designed bimolecular approach, both the click partners, dienone and dienophile (Fig. 2C), have to be conjugated with the targeting moiety, folic acid, for enrichment. We first selected BCN as the dienophile and compound **18a** as the diene. This is based on the fact that the same pair worked well after conjugation with TPP. Literature reports indicate that the α-carboxylate group of folic acid is critical for receptor binding. Therefore, the γ-carboxylate group is the preferred handle for modification.[11] To begin with, γ-carboxylate conjugated ethylene diamine folate (9) is synthesized by using a literature procedure[55] by reaction with Boc-protected ethylenediamine followed by TFA treatment to remove the Boc group. Then cyclooctyne (BCN) was conjugated through carbamylation using an activated carbonate group to provide compound **11** (Scheme 1). The γ-conjugation regiochemistry of compound **8**, **9**, and **11** is confirmed by comparing the ^1^H-NMR spectra of these compounds with the corresponding literature reports. Specifically, the ^1^H-NMR γ-folate conjugates are characterized by a singlet peak of 4.28 ppm, which is exactly matching with the literature reported NMR spectra of compound **8**, **9** and **11**.[55, 56]

For the synthesis of the folate conjugate with the cyclopentadienone partner, an ethylene diamine linker was also used (**19a-b**). To begin with, phenylacetyl chloride was reacted with Meldrum’s acid to give compound **13a-b**. Subsequent decarboxylative reaction with *t*-butyl alcohol gave compound **14**. Aldol condensation of compound **14a-b** with acenaphthylene-1,2-dione followed by dehydration in acidic medium gave compound **15a-b** in 72–79% yield. After removal of the t-Bu protective group from the ester, the carboxyl group was reacted with Boc-protected ethylene diamine using EDC, DMAP to give **17a-b**. Subsequent Boc deprotection with TFA gave cyclopentadienone tagged with an ethylene diamine linker (**18a-b**), which was conjugated with NHS activated folic acid to get compound **19a-b** (Scheme 2).

### 2.2 CO release Kinetic Studies

As discussed earlier, success of an enrichment triggered delivery approach largely depends on the reaction kinetics. As a bimolecular reaction, CO-release half-life relies on the reactivity and concentration of two individual click partners.[57, 45] In a previous study, we have already shown that a second order-rate constant of 0.2 M^− 1^s^− 1^ allows stability when dosed at single digit micromolar concentrations. However, when enriched by hundreds of folds, the reaction is proportionally accelerated to allow CO release with a half-life less than a few hours.[45] Another finding from the previous study was the improved reaction rate of the Diels-Alder reaction after conjugation. For example, the rate constant changed from 0.14 M^− 1^s^− 1^ to 0.20 M^− 1^s^− 1^ when the reaction partners were each conjugated with a TPP moiety.[45] Therefore, we first need to study the reaction kinetics after folate conjugation.

**Figure 4:**
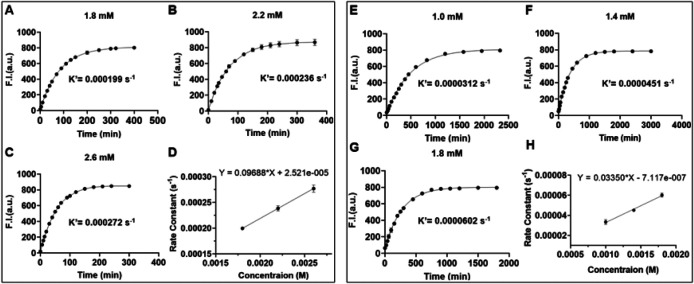
Determination of the pseudo-first order and second order rate constants for the Diels-Alder reactions. **A-C**) cycloalkyne (**21)** and cyclopentadienone (**20); E-G** cycloalkyne (**11)** and cyclopentadienone (**19b)**. Fluorescent intensity was monitored by incubating different concentrations of cyclopentadienone and a fixed concentration of cycloalkyne (50 μM) with excitation and emission wavelength of 375 and 465 nm respectively. **D, H**) Second-order rate constant between cycloalkyne and cyclopentadienone.

Our CO prodrugs are designed in such a way that the cyclization product after CO release is fluorescent and serves as way to study the kinetics for the CO release reaction.[58] Thus, the CO release kinetics by the prodrug system is studied by fluorescence spectroscopy. Briefly, BCN compound at three different concentrations are allowed to react with 50 μM of a cyclopentadienone. CO release kinetics are studied by monitoring the fluorescence at 464 nm (λ_ex_ = 375 nm), which corresponds to the formation of CO released product. A plot of fluorescence intensity against time provided the pseudo-first order rate constant for each concentration of the **BCN** compound. Further, plotting the pseudo-first order rate constant against the time provided the second order rate constant for the reaction (Fig. 4).

In determining the reaction rate constant, we first examined some “control” reactions using **20** and **21** (Fig. 4A–D). This click pair yielded a rate constants of 0.097 M^−1^s^−1^, which is generally on the same scale as the results from earlier studies of 0.14 M^−1^s^−1^ for a similar reaction.[45] Such results also gave us confidence to move forward with the synthesis of the folate conjugates. However, much to our disappointment, the reaction with 1.8 mM of BCN conjugate **11** and 50 μM of **19a** was very slow, to the extent that the fluorescence did not plateau off even after 36 h. We did not quantify the second order rate constant for the reaction between **19a** and **11** because a reaction this slow would have no practical value in our designed enrichment-triggered release approach. The slow reaction was very surprising even with the understanding of the possibly significant effects of conjugation on reaction rate. There have been literature reports of the acceleration of Diels-Alder reaction in aqueous solution due to hydrophobic effects.[59, 53] With such understanding, it is possible that the introduction of a folate moiety has substantially decreased the hydrophobicity-driven acceleration of the intended Diels-Alder reaction. With the aim of improving reaction kinetics, we designed an analog (**19b**) with a trifluoromethyl substituent to decrease the electron density of the cyclopentadienone moiety with the hope of increasing the reaction rate of this inverse-electron demand Diels-Alder reaction. When the reaction kinetics of pair of **19b** and **11** was studied, a second order rate constant of 0.033 M^−1^s^−1^ was obtained (Fig. 4E–H). Though this represents a significant improvement over **19a** and **11**, the reaction rate is still much slower than needed for the designed approach. It is also slower than the “parent” reaction without folate conjugation (0.097 M^−1^s^−1^ between **20** and **21**). With this rate constant, the t_1/2_ is calculated to be 8418 h and 842 h when incubated at 1 and 10 mM, respectively. There would need to be close to 1000-fold increase in concentration through receptor-mediated enrichment in order for the reaction rate to be in the range of meaningful CO delivery. Otherwise, such a reaction rate is not expected to be able to overcome diffusion to make a difference. Indeed, in a preliminary imaging study using cells that are Specifically designed to probe folate receptor-mediated events,[60] we were unable to see any meaningful enrichment (Fig. S1,2). Such results indicate the need for further optimization of the reaction rate. The significant difference in reaction kinetics between the two analogues underscores the significance of electronic factors of cyclopentadienone and its impact on CO release kinetics. The reaction rate difference between the folate conjugates and TPP conjugates also highlight the significant effects of the targeting moiety on reactivity. Such information combined will be helpful to future optimization work.

## 3 Conclusion

In summary, we synthesized two folate conjugated pairs for bimolecular CO prodrugs. Fluorescence studies indicate the dependence of the reaction kinetics on the electronic properties of the cyclopentadienone moiety and conjugation chemistry. Specifically, the reaction rate for p-CF_3_ substituted cyclopentadienone (**19b**) is significantly faster than the corresponding non-substituted analogue (**19a**). However, folate conjugation led to a decrease in reaction rate so much so that the presumed receptor-mediated enrichment is not sufficient to overcome the sluggish reaction rate in imaging studies. Although the design did not work out the way we envisioned, there are important lessons to learn. First, different from TPP conjugation, folate conjugation led to a decrease in reaction rate. Such findings will be very useful in guiding future designs of targeted delivery of similar reactions in terms of the effect of hydrophilic ligands on the rate of Diel-Alder reactions. Second, the enrichment magnitude of the reaction partners used is probably nowhere near the needed 1000-fold through folate receptor-mediated endocytosis. Third, it is possible to tune the reaction rate by using various electron-withdrawing groups on the dienone to speed up the reaction. Further, the search for a new strained alkyne may also lead to a solution to the current sluggish reaction issue. Overall, the results indicate the significance of reaction rate difference between targeted compound and control compound for the successful application of enrichment triggered drug delivery.

## 4 Experimental Section

### 4.1 Materials and Method

All solvents were of reagent grade and were purchased from Fisher Scientific. All chemicals are of reagent grade and purchased from Sigma-Aldrich (Massachusetts, USA) or Ambeed, Inc. (Illinois, USA) or Oakwood Products, Inc. (South Carolina, USA), Column chromatography was carried out using silica gel (Sorbent Technologies (Georgia, USA) 230–400 mesh). TLC analyses were conducted on silica gel plates (Sorbent Technologies (Georgia, USA) Silica XHL TLC plates w/UV254). ^1^H NMR (400 MHz) and ^13^C NMR (100 MHz) spectra were recorded on a Bruker Avance 400 MHz NMR spectrometer in deuterated solvent from Oakwood Products, Inc. (South Carolina, USA). Chemical shifts were reported as δ values (ppm). TMS (δ = 0.00 ppm) or residual peaks of the deuterated solvent were used as the internal reference. Mass spectrometric analyses were conducted by the Georgia State University Mass Spectrometry Facilities. The milligram scale quantities were weighed on C-33 microbalance (CAHN instruments Inc., California, USA). HPLC was performed on Shimadzu LC-20AT or Agilent HPLC system. Column: C18, 5 μm, 4.6 × 150 mm; detector: DAD monitored at 254 nm.

Mobile phase composed of A: H_2_O (0.1% TFA) and B: ACN (0.1% TFA). Gradient methods used: Analytical HPLC Method: 5% B, 0–10 min; 95% B, 10–12 min; 5% B, 12–12.1 min; 5% B, 12.1–15 min.

Preparative HPLC Method: 30% B, 0–18 min; 45% B, 18–18.1 min; 30% B, 18.1–20.0 min.

#### 4.1.1 Determination of second order rate constant for folate conjugated CO prodrug

10 mM stock solutions of compounds **19a/19b or 11** were prepared in DMSO. 50 μM of dienone (**19a/19b**) was incubated at 37 °C in 1.5 ml of cuvette with different concentration of cycloalkyne **11** (1.0 mM, 1.4 mM, 1.8 mM) in 60% DMSO:PBS The reaction progress is monitored by measuring the fluorescence intensity at 464 nm at frequent time intervals (λ_ex_= 375 nm). A plot of fluorescent intensity against time provided the pseudo first order rate constant. Next, plotting the k’ values against the different concentrations of **11** provided the second order rate constant. (Fig. 4. A–D). A similar protocol was used for the control compound dienone **20** (50 μM), but a higher concentration range of cycloalkyne **21** (1.8 mM, 2.2 mM, 2.6 mM) (Fig. 4. E–H).

#### 4.1.2 Cell-Imaging Experiment

The intracellular CO release from the folate conjugated CO prodrugs were studied by using fluorescent cell imaging. Literature reports indicate that the cell-culture in folate depleted medium helps in the FR over-expression (FR^+^ cells) and facilitates increased uptake of folate conjugates.[60]Thus, KB cells were cultured in two different media, one of the plates is cultured in normal RPMI-1540 (Gibco)) medium and other plate is cultured in folic acid depleted RPMI-1540 medium (Gibco). Both the medium is supplemented with 10% fetal bovine serum (FBS, Gibco) and 0.1% 100 × antibiotic-antimycotic solution (Gibco). To begin with, folate conjugated CO prodrug partners **11** and **19b** were co-incubated with normal KB and FR^+^-KB cells. Fluorescence images were recorded on 4 h and 16 h time point after incubation. Similarly, a control experiment is performed by incubating 4 h KB and FR^+^-KB cells with non-folate conjugated dienone **20** and cycloalkyne **21**.

#### 4.1.3 Synthetic procedure for the designed compounds

##### N2-(4-(((2-amino-4-oxo-3,4-dihydropteridin-6-yl)methyl)amino)benzoyl)-N5-(2-((tert-butoxycarbonyl)amino)ethyl)-L-glutamine (8)

Synthesized by a reported literature procedure.[55]

Folic acid was (320 mg, 0.73 mmol) was added to 8 ml of DMSO and heated at 55 °C for 30 min to get a clear solution. The solution was cooled to room temperature and added N-hydroxy succinimide (167 mg, 1.46 mmol) and DCC (301 mg, 1.46 mmol) under nitrogen atmosphere and protected from light, stirred at room temperature for 20 h. After 20 h of stirring, the urea precipitate was filtered off and the obtained solution is used for the next step by adding triethylamine (203 μL, 1.46 mmol) followed by N-Boc-ethylenediamine (234 mg, 1.46 mmol) and stirred for 16 h. Once the reaction is completed, 20% acetone in diethyl ether solution is added to the reaction mixture and the resulting light-yellow precipitate is collected with the aid of centrifugation. The HPLC analysis of the reaction mixture indicates a product formation with a new peak with almost 20% of folic acid remain unreacted which was further purified by preparative HPLC (Shimadzu HPLC-0.1% TFA in ACN/ H20; Method 0–18 min 30%−45%, flowrate 16 ml/min). The purity of the obtained product was confirmed by recording the ^1^H NMR and matching the literature reports[56, 55] The compound isolated as yellow solid, 267 mg, yield: 63 %. ^1^H NMR (DMSO- *d6*) δ 11.52 (s, 1H), 8.66 (d, *J* = 15.1 Hz, 1H), 8.04 (d, *J* = 7.1 Hz, 1H), 7.86 (d, 1H), 7.70 (dd, 8.5 Hz, 2H), 6.96 (s, 2H), 6.74 (dd, *J* = 11.1 Hz, 2H), 6.63 (d, *J* = 8.6 Hz, 2H), 4.52 (s, 2H), 4.28 (s, 1H), 3.15 – 3.02 (m, 2H), 2.96 (s, 2H), 2.19 – 1.78 (m, 4H), 1.35 (d, *J* = 6.1 Hz, 9H).

##### N2-(4-(((2-amino-4-oxo-3,4-dihydropteridin-6-yl)methyl)amino)benzoyl)-N5-(2-aminoethyl)-L-glutamine (9)

Synthesized by using a previous literature procedure.[55]

Compound **8** (200 mg, 0,34 mmol) was dissolved in 5 ml of TFA and stirred at room temperature for 3 h, the reaction progress was monitored by HPLC and once the reaction is completed, the excess TFA is evaporated with the aid of 5 ml DCM and the resulting content of the flask is dissolved in 2 ml of DMF, and the product is precipitated by the slow addition of triethylamine (~ 700 μL). The light-yellow solid precipitate is collected by centrifugation to obtain compound **9** with quantitative yield. This product is used for the subsequent conjugation steps with CO prodrug partners.^1^H NMR (DMSO- *d6*) δ 8.61 (s, 1H), 8.10 (s, 1H), 7.96 (s, 2H), 7.66 (d, *J* = 6.9 Hz, 2H), 7.17 (s, 2H), 6.96 (s, 1H), 6.64 (d, *J* = 7.7 Hz, 2H), 4.48 (s, 2H), 4.28 (s, 1H), 3.13 (s, 2H), 3.11 (s, 2H), 2.63 (s, 2H), 2.12 (d, 2H), 2.12 – 1.92 (m, 2H), 1.84 (d, *J* = 10.3 Hz, 2H).

##### ((1R,8S,9s)-bicyclo[6.1.0]non-4-yn-9-yl)methyl (4-nitrophenyl) carbonate (10)

((1R,8S,9s)-bicyclo[6.1.0]non-4-yn-9-yl)methanol (50 mg, 0.33mmol) and pyridine (107 μL, 1.3 mmol) was dissolved in DCM and stirred at room temperature for 10 minutes. To this solution 4-nitrophenyl chloroformate (102 mg, 0.5 mmol) was added and stirred at room temperature for 4 h under nitrogen atmosphere. The reaction mixture is worked up with 0.1 M HCl for three times and collected organic layer is dried over anhydrous Na_2_SO_4_ and concentrated in vacuo. The crude product was purified by silica gel column chromatography (Hexane: Ethyl acetate = 80:20) to afford compound **10** as pure white solid, 60 mg, yield: 57 %. ^1^H NMR (CDCl_3_) δ 8.30 (d, J = 9.2 Hz, 2H), 7.37 (t, 2H), 4.42 (d, J = 8.3 Hz, 2H), 2.64 – 2.13 (m, 6H), 1.70 – 1.45 (m, 3H), 1.22 – 0.90 (m, 2H); ^13^C NMR (CDCl_3_) δ 155.6, 152.6, 145.4, 125.3, 121.8, 98.7, 68.0, 29.0, 21.4, 20.5, 17.2; HRMS (ESI^+^) m/z calcd for C_17_H_17_NO_5_Na 338.1004, found 338.1009 [M+Na]^+^.

##### N2-(4-(((2-amino-4-oxo-3,4-dihydropteridin-6-yl)methyl)amino)benzoyl)-N5-(2-(((((1R,8S,9s)-bicyclo[6.1.0]non-4-yn-9-yl)methoxy)carbonyl)amino)ethyl)-L-glutamine (11)

Synthesized by using a previous literature procedure.[55]

To a solution of compound **9** (25 mg, 0.052 mmol) in 1.5 ml anhydrous DMSO, was added NN-Diisopropylethylamine (27 μL, 0.156 mmol) and compound **10** (18 mg, 0.057 mmol) and stirred for 1.5 h to get a bright-yellow colored solution. The reaction mixture was added to a solution of 20% acetone in diethyl ether and the resulting light-yellow precipitate is collected by centrifugation. The resulting precipitate is washed three times with acetone and diethyl ether and the pure precipitate is collected by centrifugation and air dried for 24 h. The purity of the obtained product was confirmed by recording the ^1^H NMR and matching the literature reports.[56, 55] The product is isolated as a yellow solid, 29 mg, yield: 86%.^1^H NMR (DMSO-*d6*) δ 8.63 (s, 1H), 8.04 (s, 1H), 7.90 (s, 1H), 7.67 (s, 2H), 7.09 (d, *J* = 13.1 Hz, 1H), 6.96 (s, 2H), 6.63 (d, *J* = 8.5 Hz, 2H), 4.48 (s, 2H), 4.28 (s, 1H), 4.08 – 3.92 (m, 2H), 3.09 (s, 2H), 3.01 (s, 2H), 2.04 (dt, 10H), 1.51 (s, 2H), 1.24 (s, 2H), 0.84 (s, 2H).

##### 6-hydroxy-2,2-dimethyl-5-(2-phenylacetyl)-4H-1,3-dioxin-4-one (13a)

Synthesized by a previously reported literature procedure.[45]

To a solution of Meldrum’s acid (1.0 g, 6.9 mmol) and pyridine (1.1 mL, 13.8 mmol) in dichloromethane (15 ml) under ice-cold temperature was added a solution of phenylacetyl chloride (1.278 g ,8.3 mmol.) drop by drop. After the completion of addition, the reaction was allowed to stir at room temperature, and stirred for an additional 3 h. The reaction mixture was then washed successively with 5% HCl solution and brine. The organic layer was dried with anhydrous Na_2_SO_4_, filtered and concentrated. The obtained residue was purified by silica gel column chromatography (Hexane: ethyl acetate = 7:3) to afford compound **13a** as white solid,1030 mg, yield: 57 %.

##### tert-butyl 3-oxo-4-phenylbutanoate(14a)

Compound **13a** (500 mg, 1.90 mmol) was dissolved in 8 ml of *tert*-butanol and refluxed at 85 °C for 3 h. Upon completion of the reaction, the content of the flask was evaporated to dryness to obtain yellow viscous liquid with quantitative yield (422 mg). ^1^H NMR (CDCl_3_) δ 7.38 – 7.29 (m, 3H), 7.22 (d, *J* = 7.2 Hz, 2H), 3.82 (s, 2H), 3.39 (s, 2H), 1.48 (s, 10H); ^13^C NMR (CDCl_3_) δ 200.9, 166.4, 133.5, 129.7, 128.8, 127.3, 81.9, 49.9, 49.6, 28.3, 28.0; HRMS (ESI^+^) m/z calcd for C_14_H_19_O_3_ 235.1334, found 235.1336 [M+H]^+^.

##### tert-butyl 8-oxo-9-phenyl-8H-cyclopenta[a]acenaphthylene-7-carboxylate (15a)

A solution of compound **14a** (400 mg 1.71 mmol), and acenaphthylene-1, 2-dione (373 mg, 2.05 mmol.) in THF/MeOH (3/1, v/v, 12 ml) was treated with Et_3_N (362 μL, 2.6 mmol). Then the mixture was stirred at room temperature for 3 h, after which the mixture was concentrated under vacuum, and the resulting residue was dissolved in acetic anhydride (4 mL). The resulting solution was cooled to 0 °C, and 2 drops of concentrated sulfuric acid was added. The reaction mixture was stirred for an additional 10 min at 0 °C and the reaction mixture was diluted with ethyl acetate (30 ml) and washed with NaHCO_3_ solution. The organic layer was dried over anhydrous Na_2_SO_4_. After concentration, the residue was purified over silica gel column (Hexane: Ethyl acetate 99: 1) to yield compound **15a** as purple solid, 467 mg, yield: 72%.^1^H NMR (CDCl_3_) δ 8.77 (d, *J* = 7.1 Hz, 1H), 8.04 (dd, *J* = 7.4, 5.7 Hz, 2H), 7.92 (d, *J* = 8.2 Hz, 1H), 7.84 – 7.72 (m, 3H), 7.66 – 7.59 (m, 1H), 7.54 (t, *J* = 7.5 Hz, 2H), 7.46 (d, *J* = 7.3 Hz, 1H), 1.70 (s, 9H); ^13^C NMR (CDCl_3_) δ 197.3, 168.4, 161.9, 150.8, 144.9, 131.5, 130.7, 130.4, 130.4, 130.1, 129.6, 128.9, 128.8, 128.6, 128.3, 127.9, 127.7, 123.60, 121.1, 111.8, 81.5, 28.5; HRMS (ESI^+^) m/z calcd for C_26_H_21_O_3_ 381.1491, found 381.1501 [M+H]^+^.

##### 8-oxo-9-phenyl-8H-cyclopenta[a]acenaphthylene-7-carboxylic acid (16a)

A solution of compound **15a** (450 mg, 1.18 mmol) in 3 ml dichloromethane was cooled to 0 °C, to which was added trifluoroacetic acid (12 ml) as dropwise and allowed to stir for 2 h. The reaction mixture was concentrated in vacuo and used for the next step without further purification (363 mg).

##### tert-butyl(2-(N-methyl-8-oxo-9-phenyl-8H-cyclopenta[a]acenaphthylene-7-carboxamido)ethyl)carbamate (17a)

To a solution of compound **16a** (325 mg, 1.0 mmol) in DCM, was added EDC (288 mg, 1.5 mmol) and DMAP (183 mg, 1.5 mmol) and tert-butyl (2 (methylamino)ethyl) carbamate (261mg, 1.5 mmol) andallowed to stir at room temperature for overnight. Once the reaction is completed, the reaction mixture is diluted with DCM and washed with 0.1 N HCl for 3 times, followed by 3 times with brine and the organic layer is collected and dried over anhydrous Na_2_SO_4_ and concentrated in-vacuo and purified by silica gel flash column chromatography (Hexane: Ethyl acetate (95:5) to get the compound **17a** as reddish brown solid, 404 mg, yield: 84%.^1^H NMR (CDCl_3_) δ 7.95 (dd, *J* = 10.8, 7.2 Hz, 2H), 7.89 (d, *J* = 8.1 Hz, 1H), 7.82 (d, *J* = 8.2 Hz, 1H), 7.75 (d, *J* = 7.5 Hz, 2H), 7.62 (t, *J* = 7.6 Hz, 1H), 7.57 – 7.44 (m, 3H), 7.39 (t, *J* = 7.3 Hz, 1H), 5.49 (s, 1H), 3.72 (d, *J* = 5.3 Hz, 2H), 3.56 – 3.41 (m, 2H), 3.11 (s, 3H), 1.45 (s, 9H); ^13^C NMR (CDCl_3_) δ 198.8, 165.2, 162.8,156.3,151.9, 145.1, 131.8, 130.8, 130.7, 129.9, 129.4, 129.2, 128.8, 128.7, 128.5, 127.9, 124.8, 122.4, 121.2, 115.8,79.3, 46.9, 38.9, 37.3, 28.5; HRMS (ESI^+^) m/z calcd for C_30_H_29_N_2_O_4_ 481.2127, found 481.2119 [M+H]^+^.

##### N-(2-aminoethyl)-N-methyl-8-oxo-9-phenyl-8H-cyclopenta[a]acenaphthylene-7-carboxamide (18a)

A solution of compound **17a** (200 mg, 0.42 mmol) in DCM (3 ml) was cooled to 0 °C, to which was added trifluoroacetic acid (6 ml) as dropwise and allowed to stir for 2 h. The reaction mixture was concentrated in vacuo with the aid of DCM and used for the next step without further purification.

##### N2-(4-(((2-amino-4-oxo-3,4-dihydropteridin-6-yl)methyl)amino)benzoyl)-N5-(2-(N-methyl-8-oxo-9-phenyl-8H-cyclopenta[a]acenaphthylene-7-carboxamido)ethyl)glutamine (19a)

To a solution of compound **7** (25 mg, 0.05 mmol) in DMSO was added triethylamine (11.4 μL, 0.08 mmol) and followed by compound **18a** (31 mg, 0.08 mmol) and stirred at room temperature for 30 min. The product is precipitated as a dark brown precipitate by the addition of 20% acetone in diethyl ether, which was washed three times with diethyl ether and three times with acetone and collected by the aid of centrifugation to get compound **19a** as reddish brown solid, 24 mg, yield: 54%. HRMS (ESI^−^) m/z calcd for HRMS calculated for C_44_H_36_N_9_O_7_ 802.2738, found 802.2737 [M-H]^+^. Further confirmed by LC-MS.

###### 6-hydroxy-2,2-dimethyl-5-(2-(4-(trifluoromethyl)phenyl)acetyl)-4H-1,3-dioxin-4-one (13b):

To a solution of Meldrum’s acid (1.0 g, 6.9 mmol) and pyridine (1.1 mL, 13.8 mmol) in dichloromethane (15 ml) under ice-cold temperature was added a solution of 2-(4-(trifluoromethyl)phenyl)acetyl chloride (1.84 g ,8.3 mmol.) drop by drop. After the completion of addition, the reaction was allowed to stir at room temperature, and stirred for an additional 6 h. The reaction mixture was then washed successively with 5% HCl solution and brine. The organic layer was dried with anhydrous Na_2_SO_4_, filtered and concentrated. The obtained residue was purified by silica gel column chromatography (Hexane: ethyl acetate = 7:3) to afford compound **13b** as white solid,1297 mg, yield: 59 %. ^1^H NMR (CDCl_3_) δ 15.40 (s, 1H), 7.61 (d, *J* = 8.2 Hz, 2H), 7.53 (d, *J* = 8.2 Hz, 2H), 4.49 (s, 2H), 1.74 (d, *J* = 5.8 Hz, 6H). ^13^C NMR (CDCl_3_) δ 193.3, 170.5, 160.3, 138.0, 130.0, 129.7, 125.7, 122.7, 105.3, 91.8, 40.6, 26.9. HRMS (ESI^−^) m/z calcd for C_15_H_13_F_3_O_5_ 329.0637, found 329.0639 [M-H]^+^.

###### tert-butyl 3-oxo-4-(4-(trifluoromethyl)phenyl)butanoate(14b):

Compound **13b** (660 mg, 2 mmol) was dissolved in 10 ml of t-butanol and the reaction was refluxed for 4 h at 85 °C. The reaction mixture was concentrated in vacuo to give compound **14b** as slight yellow viscous liquid, 565 mg, yield: 92%.^1^H NMR (CDCl_3_) δ 7.61 (d, *J* = 8.1 Hz, 2H), 7.34 (d, *J* = 8.0 Hz, 2H), 3.93 (s, 2H), 3.43 (s, 2H), 1.48 (s, 9H). ^13^C NMR (CDCl_3_) δ 199.9, 166.1, 137.3, 130.1, 129.7, 125.6, 122.8, 82.4, 50.0, 49.3, 28.0. HRMS (ESI^+^) m/z calcd for C_15_H_18_F_3_O_3_ 303.1208, found 303.1215 [M+H]^+^.

###### tert-butyl8-oxo-9-(4-(trifluoromethyl)phenyl)-8H-cyclopenta[a]acenaphthylene-7-carboxylate (15b):

To a solution of compound **14b** (565 mg, 1.87 mmol), and acenaphthylene-1, 2-dione (373 mg, 2.05 equiv.) in 10 ml DMF was added triethylamine (390 μL, 2.8 mmol). Then the mixture was stirred at room temperature for 3 h, after which the mixture was concentrated under vacuum, and the resulting residue was dissolved in acetic anhydride (4 ml). The resulting solution was cooled to 0 °C, and 2 drops of concentrated H_2_SO_4_ was added. The reaction mixture was stirred for an additional 10 min at 0 °C, The reaction mixture was diluted with ethyl acetate (30 ml), and washed with water. The organic layer was dried over anhydrous Na_2_SO_4_. After concentration, the residue was purified over silica gel column (Hexane: Ethyl acetate 99: 1) to yield compound **15b** as pure purple solid 603 mg, yield: 79%.^1^H NMR (CDCl_3_) δ 8.41 (d, *J* = 7.1 Hz, 1H), 7.84 – 7.62 (m, 7H), 7.52 (t, *J* = 7.7 Hz, 1H), 7.40 (t, *J* = 7.6 Hz, 1H), 1.70 (s, 9H).^13^C NMR (CDCl_3_) δ 196.3, 167.5, 161.5, 152.4, 144.8, 134.2, 131.4, 130.4, 130.3, 129.67, 129.6, 129.5, 128.8, 128.3, 128.2, 128.0, 125.4, 122.7, 121.4, 121.3, 112.0, 81.7, 28.5. HRMS (ESI^+^) m/z calcd for C_27_H_19_F_3_O_3_ 449.1365, found 449.1361[M+H]^+^.

###### 8-oxo-9-(4-(trifluoromethyl)phenyl)-8H-cyclopenta[a]acenaphthylene-7-carboxylic acid(16b):

A solution of compound **15b** (0.526 mmol) in 5 ml dichloromethane was cooled to 0 °C, to which was added trifluoroacetic acid (0.2 ml) as dropwise and allowed to stir for 2 h. The reaction mixture was concentrated in vacuo and used for the next step without further purification.

##### tert-butyl(2-(N-methyl-8-oxo-9-(4-(trifluoromethyl)phenyl)-8H-cyclopenta[a]acenaphthylene-7-carboxamido)ethyl)carbamate (17b).

To a solution of compound **16b** (300 mg, 0.76 mmol) in DCM, was added EDC (220 mg, 1.14 mmol), DMAP (139 mg, 1.14 mmol) and followed by tert-butyl (2 (methylamino)ethyl) carbamate (198 mg, 1.14 mmol). The reaction mixture was allowed to stir at room temperature overnight. Once the reaction is completed, the reaction mixture is diluted with DCM and washed with 0.1 N HCl for 3 times, followed by 3 times with brine and the organic layer is collected and dried over anhydrous Na_2_SO_4_ and concentrated in-vacuo and purified by silica gel column chromatography (Hexane: Ethyl acetate (99: 1) to get the compound **17b** as purple solid, 350 mg, yield: 88%. ^1^H NMR (CDCl_3_) δ 7.94 (m, *J* = 20.1 Hz, 6H), 7.77 (d, *J* = 8.1 Hz, 2H), 7.68 (dd, *J* = 9.7, 5.5 Hz, 1H), 7.60 (t, *J* = 7.5 Hz, 1H), 5.45 (s, 1H), 3.75 (d, *J* = 5.6 Hz, 2H), 3.52 (d, *J* = 16.9, 2H), 3.14 (s, 3H), 1.48 (s, 9H). ^13^C NMR (CDCl_3_) δ 198.2, 164.9, 162.3, 156.3, 154.0, 145.3, 134.4, 131.9, 130.4, 129.6,129.6, 129.4, 129.0, 128.6, 125.6, 125.0, 122.7, 121.6, 120.7, 116.2, 79.5, 46.9, 38.8, 37.2, 28.8. HRMS (ESI^+^) m/z calcd for C_31_H_28_F_3_N_2_O_4_ 549.2001; found 549.2028 [M+H]^+^.

###### N-(2-aminoethyl)-N-methyl-8-oxo-9-(4-(trifluoromethyl)phenyl)-8H-cyclopenta[a]acenaphthylene-7-carboxamide (18b):

Compound **17b** (350 mg, 0.64 mmol) was dissolved in 10 ml solution of DCM: TFA (1:2), and was stirred under ice bath for 2 h. After completing the reaction, the contents of the flask was evaporated and the product obtained is used for the next step without further purification.

###### N2-(4-(((2-amino-4-oxo-3,4-dihydropteridin-6-yl)methyl)amino)benzoyl)-N5-(2-(N-methyl-8-oxo-9-(4-(trifluoromethyl)phenyl)-8H-cyclopenta[a]acenaphthylene-7-carboxamido)ethyl)glutamine (19b):

To a solution of compound 7 (25 mg, 0.05 mmol) in DMSO was added triethylamine (11.4 μL, 0.08 mmol) and followed by compound **18b** (36 mg, 0.08 mmol) and stirred at room temperature for 30 min. The product is precipitated as a dark brown precipitate by the addition of 20% acetone in ethyl ether, which was washed three times with diethyl ether and three times with acetone and collected by the aid of centrifugation to get compound **19b** as brown solid, 24 mg, yield: 58 %. HRMS (ESI^−^) m/z calcd for HRMS calculated for C_45_H_35_F_3_N_9_O_7_ 870.2612, found 870.2617 [M-H]^+^. Further confirmed by LC-MS.

###### N-ethyl-N-methyl-8-oxo-9-(4-(trifluoromethyl)phenyl)-8H-cyclopenta[a]acenaphthylene-7-carboxamide (20)

To a solution of compound **16b** (200 mg, 0.51 mmol) in DCM was added (85 μL, 1.02 mmol) of oxalyl chloride and 2 drop of DMF, allowed to stir at room temperature in inert atmosphere for 2 h. Once the reaction is completed the contents of the flask was evaporated to dryness and added N-methylethanamine (174 μL, 1.02 mmol), triethylamine (145 μL, 1.02 mmol) and stirred at room temperature for 1.5 h. On reaction completion, the reaction mixture was washed 3 times with 0.1 N HCl and brine. The organic layer was collected and dried over anhydrous Na_2_SO_4_ and concentrated in vacuo, purified by silica gel column chromatography (Hexane: Ethyl acetate (90: 10) to obtain a purple solid 139 mg, yield: 63%. ^1^H NMR (CDCl_3_) δ 7.67–8.05(m, 2H), 7.73 (d, J=8.0 Hz, 4H), 7.66–7.70(m, 2H), 7.62(t, *J* = 8.2 Hz, 2H), 3.66 (q, 2H), 3.45 (q, 2H), 3.16 (s, 3H), 3.09 (s, 3H), 1.31(t, 3H), 1.22(t, 3H). ^13^C NMR (CDCl_3_) δ 198.38, 163.83, 161.0, 154.00, 145.25, 134.51, 131.95, 130.51,130.28, 129.83, 129.4, 129.3, 128.9, 128.6, 125.4, 124.5,122.71, 121.5, 120.7, 116.9, 45.9, 42.3, 36.1, 32.17, 14.0, 12.3. HRMS (ESI^+^) m/z calcd for C_26_H_19_F_3_NO_2_ 434.1368, found 434.1363 (M+H)^+^.
